# Tailored Individual Follow-Ups Versus a One-Day Group Course in Patients With Long COVID (Post– COVID-19 Condition): Protocol for a Randomized Controlled Trial

**DOI:** 10.2196/74113

**Published:** 2026-01-05

**Authors:** Marte Wilson, Synne Garder Pedersen, Nina Langeland, Rebecca Jane Cox, Pål Aukrust, Tuva B Dahl, Axel Sandvig, Maja Wilhelmsen

**Affiliations:** 1 Department of Rehabilitation, University Hospital of North Norway, Tromsø, Norway; 2 Institute of Health and Society, Research Centre for Habilitation and Rehabilitation Model and Services (CHARM), Faculty of Medicine, University of Oslo, Oslo, Norway; 3 Department of Clinical Science, University of Bergen, Bergen, Norway; 4 Department of Medicine, Haukeland University Hospital, Bergen, Vestland, Norway; 5 Norwegian Institute for Public Health, Oslo, Norway; 6 Influenza Centre, Department of Microbiology, Haukeland University Hospital, Bergen, Vestland, Norway; 7 Research Institute of Internal Medicine, Rikshospitalet, Oslo University Hospital, Oslo, Norway; 8 Faculty of Medicine, University of Oslo, Oslo, Norway; 9 Department of Neuromedicine and Movement Science, Norwegian University of Science and Technology, Trondheim, Norway; 10 Department of Clinical Medicine, UiT The Arctic University of Norway, Postboks 6050 Stakkevollan, Tromsø, 9037, Norway, +47 99001559

**Keywords:** postacute COVID-19 syndrome, graded activity, fatigue, rehabilitation, cognitive training, telerehabilitation, randomized controlled trial, cognitive behavioral therapy, exercise, mobile apps

## Abstract

**Background:**

The high prevalence of patients with post–COVID-19 condition, also called long COVID, even among those with mild initial disease, may have a large impact on both the individual and society. Disability in everyday life, reduced health-related quality of life and work capacity, strain on the health care system, and substantial socioeconomic costs are associated with long COVID. More research to investigate the effectiveness of rehabilitation services is warranted.

**Objective:**

This study aims to examine the effectiveness of tailored individual follow-ups versus a 1-day group course in patients with long COVID. Additionally, the feasibility and use of a mobile app for self-monitoring goal achievement will be assessed.

**Methods:**

This is a single-center, parallel-group, superiority randomized controlled trial with a 1:1 allocation ratio. A total of 62 outpatients aged 18‐65 years with long COVID will be randomized to either a rehabilitation program with individual follow-up consultations or a 1-day self-management group course. The individual intervention incorporates setting goals, teaching cognitive behavioral strategies, energy management (pacing), and a supervised gradual increase in both physical and cognitive activities tailored to individual tolerance levels. The primary outcome is the between-group difference in health-related quality of life, measured using the EQ-5D-5L index at 6 months. Secondary outcomes include improvements in symptoms, work participation, neurocognitive function, and app usability, assessed at 3, 6, and 12 months, depending on the outcome measure.

**Results:**

Data enrollment started in October 2023. A total of 62 participants were included by November 2024. Data collection is planned to be completed in November 2025.

**Conclusions:**

Long COVID poses substantial challenges for both individuals and society, underscoring the need for effective rehabilitation strategies. This study will provide valuable insights into the benefits of an individualized outpatient rehabilitation program. The results from this clinical trial will help guide future treatment recommendations and may improve long-term outcomes for affected patients. Additionally, the study will generate important knowledge about neuropsychological function and digital self-management tools in long COVID rehabilitation.

## Introduction

### Symptoms and Consequences

By the end of 2023, more than 277 million confirmed SARS-CoV-2 infections were reported in Europe [1]. According to the World Health Organization (WHO), 10% to 20% of those who have been infected develop post–COVID-19 condition, also called long COVID. This condition is defined as “individuals with a history of probable or confirmed SARS-CoV-2 infection, usually 3 months from the onset, with symptoms that last for at least 2 months and cannot be explained by an alternative diagnosis. Common symptoms include, but are not limited to, fatigue, shortness of breath, and cognitive dysfunction, and generally have an impact on everyday functioning” [2]. Long-lasting symptoms affect not only hospitalized patients, but worryingly also nonhospitalized people after a mild infection [3].

Long COVID is a complex multisystem secondary condition [4] and a new disease of partly unknown pathogenesis [5-7]. Long-term symptoms following infection are similar to those found in conditions where the central nervous system is affected [5,6] and consist of key symptoms similar to chronic fatigue syndrome/myalgic encephalomyelitis (CFS/ME) [8,9]. One of the main complaints reported by patients with long COVID is impaired cognitive function [9-13], which includes self-reported fatigue, memory deficits, difficulty concentrating, and difficulty in information processing. A variety of other persistent symptoms are reported, including headache, dyspnea, cough, chest pain, myalgia, joint pain, impaired mobility, sleep disorders, depression, and anxiety [10]. Symptoms of long COVID may lead to disability in everyday life, reduced health-related quality of life (HRQoL) [14,15], strain on the health care system, and substantial socioeconomic costs [15]. Moreover, long COVID is strongly associated with a reduction in workability [16].

### Treatment Content

Different treatment options are delivered to patients with long COVID: drug interventions, dietary interventions, or behavioral interventions [17]. In patients with complex illness, behavioral interventions are often an important factor stimulating recovery [18,19]. Behavioral interventions can vary in intensity (eg, number of consultations), content (eg, graded activity or aerobic training), modality (eg, group-based or individual), and may be mono- or multidisciplinary.

Cognitive behavioral therapy (CBT) is based on the theory that our body (symptoms), thoughts, feelings, and behaviors are interconnected. Adjustments in behavior and thoughts can be helpful for somatic symptoms, and adjustments can be implemented through home-based exercises [18]. Results of a randomized controlled trial (RCT) using CBT have shown significantly less severe fatigue in patients with long COVID across follow-up assessments compared to patients receiving standard care [20]. Participants received substantial follow-ups over 17 weeks, and the control group received care as usual. All secondary outcomes, including social and physical functioning, somatic symptoms, and concentration, also favored patients receiving CBT; however, HRQoL was not evaluated. Patients in this study were mostly nonhospitalized [20]. A recent review recommends behavioral interventions based on CBT as management of long COVID [17].

A previous systematic review reported that regular physical exercises increase physical performance, neurocognitive function as well as fatigue and HRQoL [3] in patients with long COVID. In a cohort study by Daynes et al [21], patients with long COVID showed improvements in all those parameters listed above after supervised exercise training (aerobic and strength training) in combination with education and pacing advice twice a week for 6 weeks. However, this study had only 30 participants and did not include a control group.

A case-control study by Palladini et al [22] detected improved cognitive performance in hospitalized patients with long COVID, with a correlation to measures of quality of life. The study intervention included cognitive exercises of the most affected cognitive domains and counseling follow-ups for 2 months. Furthermore, cognitive exercise in patients with long COVID is supported by several other studies [5,9,23].

According to the WHO, patients with long COVID may experience worsening of symptoms following minimal cognitive, physical, emotional, and social activity, or activity that could previously be tolerated [24]. A result of this is often a behavioral challenge where patients exceed their own tolerance limit, followed by complete rest, an all-or-nothing pattern. Others may generally fear activity because it may lead to exacerbation of symptoms, also called fear-avoidance. Such patterns are often seen in CFS/ME [25]. Graded fixed incremental exercise therapy is debated in the literature because it might cause PEM, which is associated with poor health outcomes [26-29]. Pacing, on the other hand, is how to balance activities and rest to avoid exacerbation of symptoms, and thus not fixed incremental steps [24]. In patients with long COVID, individualized activity plans with education on pacing should therefore be provided. It is documented as beneficial to gradually introduce activity to restore patients to previous levels of activity, including daily routines [5,30]. Interventions that combine pacing and graded escalation of physical and cognitive activities are called graded activity [25]. A recent review (2023) found graded activity based on elements from CBT to be effective in reducing fatigue and improving physical functioning in CFS/ME [25].

Findings from previous studies indicate that long COVID is a complex condition that can be treated by a graded activity intervention. Daily routines and regular physical and cognitive exercise may be 3 important focus areas in this behavioral management of long COVID.

### Treatment Modality

Evidence from recent studies supports international guidelines [24,31] in terms of individual [17], multidisciplinary, and multimodal [15,21,32] rehabilitation approaches in long COVID.

In patients with CFS/ME, a self-management program delivered as group education without follow-up consultations did not have any effect compared to usual care on fatigue and physical functioning [33]. In contrast, a recent review on CFS/ME found that individual clinical follow-ups have an effect on fatigue and physical functioning [25]. Further, a digital individual self-efficacy program based on a holistic approach showed several benefits in supporting physical health and mental well-being in patients with long COVID [34].

Telerehabilitation, including video consultations, has also shown positive results in long COVID [35-37]. To ensure continuity in the rehabilitation process and promote self-management of individual rehabilitation goals [38], mobile apps have also shown effectiveness in long COVID [37,39,40].

### Knowledge Gap and Study Aims

Until today, rehabilitation studies in patients with long COVID are limited and show a substantial heterogeneity in treatment content, modality, and outcome measures, making it difficult to conclude the effectiveness [3,15,17]. Common to many interventions is that they provide clinical follow-ups over time, from 6 to 17 weeks [3,15,20,22,35]. It is still unclear how treatment content should be combined and which modality is most effective.

Recent comprehensive reviews propose conducting high-quality RCT studies to gain more insight into strategies for structuring the rehabilitation and examining the effectiveness of the management of long COVID [3,9,15,17,41]. It is unclear what treatment option is best to achieve the goal of restoring patients with long COVID to their previous levels of functioning [3], everyday function, including HRQoL [15], cognitive function [9], and workability [5-7]. More research is warranted to evaluate cognitive symptoms in patients with long COVID, and most conducted studies have used self-reported outcome measures [8,10]. Supplementing assessments with standardized neuropsychological tests may refine research findings.

To the best of our knowledge, no RCTs have so far investigated the management of long COVID with neurocognitive complaints comparing 2 rehabilitative interventions: a graded activity intervention with tailored individual follow-ups versus a one-day group course. Both intervention groups will be recommended to use the mobile app *My COVID Rehabilitation* as a supplement to the intervention for self-management of personal goals. Our RCT addresses the present knowledge gap, aiming to add robust evidence on what may be an effective rehabilitation approach for long COVID. The main research question is do tailored individual follow-ups give better outcomes in patients with long COVID compared to a 1-day group course? Additionally, the study will generate important knowledge about neuropsychological function and a digital self-management tool in long COVID rehabilitation.

## Methods

This study protocol adheres to the SPIRIT (Standard Protocol Items: Recommendations for Interventional Trials) guidelines (Checklist 1), providing reporting recommendations for protocols of randomized trials [42].

### Study Setting

This is a single-center, two-armed, parallel-group, superiority RCT with a 1:1 allocation ratio. Assessments are at baseline and at 3, 6, and 12 months. Participants will receive either individual rehabilitation (intervention group) or a 1-day group course (standard treatment). The study is embedded in ordinary clinical care at the University Hospital of North Norway (UNN). No blinding is performed except for a blinded statistical evaluation.

### Participants

A total of 62 adult participants will be included in this trial at the outpatient regional long COVID clinic at UNN. Patients are first thoroughly examined by their general practitioners to exclude other underlying diseases explaining current symptoms. Patients with symptoms of long COVID are referred to our regional long COVID clinic where clinical assessment is done primarily in a video consultation. The assessment is done by a bi-disciplinary team led by a doctor, supplemented by either a physiotherapist or a psychologist based on the medical history. During the video consultation, patients are briefly informed about the study, and consent for a SMS text message invitation is obtained.

### Eligibility Criteria

Individuals will be included if they had (1) symptoms attributable to long COVID affecting daily activities according to the WHO’s definition, (2) a positive COVID-19 test via a home test or polymerase chain reaction test, (3) neurocognitive symptoms, (4) age between 18 and 65 years, and (5) ability and willingness to provide informed consent. Exclusion criteria were as follows: (1) patients who did not want to comply with the planned physical study visits, (2) patients who were unable to complete surveys in Norwegian, and (3) patients with known chronic neurocognitive disease before COVID-19 or other diseases that can explain current symptoms. The inclusion age is set to 18 to 65 years, as one of the outcome measures in the study is return to work.

After assessment in the COVID clinic, eligible patients will receive an SMS text message invitation with a link to an information sheet and consent form (Multimedia Appendix 1). Patients are encouraged to contact the research coordinator by phone for supplementary information and possibly provide oral consent. Written informed consent is collected at the first visit. Each participant has the right to withdraw from the study at any time. Any adverse events will be recorded. The research coordinator is responsible for the enrollment process.

### Randomization

**Figure 1.**
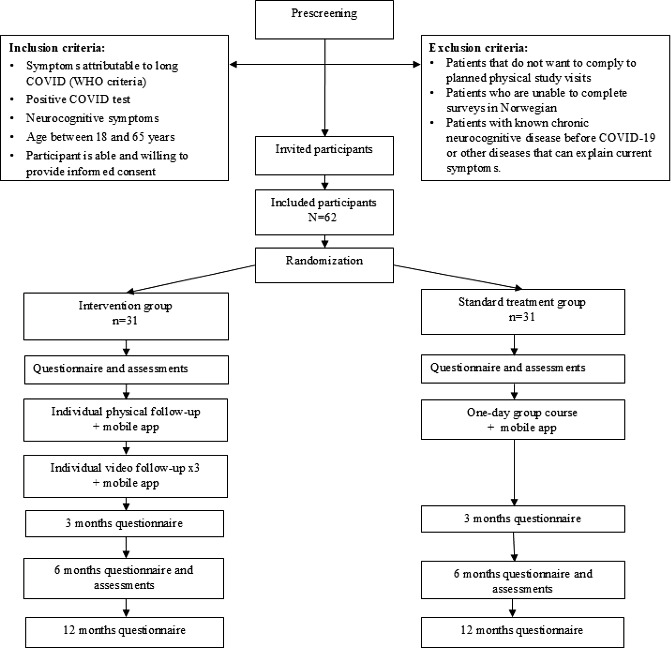
Study design and overview of data collection. WHO: World Health Organization.

Participants will be block randomized with the computer program Research Electronic Data Capture (REDCap) either to the intervention group (n=31) or standard treatment group (n=31), presented in Figure 1.

### Standard Treatment: One-Day Group Course

Standard treatment at UNN is a one-day multidisciplinary group course. The duration is 6 hours, including breaks. Each group consists of 8 to 10 participants. The aim of the course is to improve self-management of long COVID. The course provides education about (1) sustaining factors of long COVID based on elements from a CBT framework, (2) activity regulation, (3) the importance of daily routines, and the advantages of both (4) physical and (5) cognitive exercise. Information is provided by a doctor, a psychologist, and an occupational therapist or a physiotherapist. Exchange of experience between participants is also facilitated. No follow-up consultations are provided. Information about the mobile app supplement is offered; a mobile app called *My COVID Rehabilitation*. The participants can voluntarily download the mobile app as a supplement, and personal goals can be noted and adjusted.

### Study Intervention

The intervention in this study is delivered individually by a physiotherapist following the study instructions described in Textbox 1. The physiotherapist may consult the multidisciplinary team if necessary. The intervention begins with a physical consultation where symptoms, function in everyday life, sustaining factors, and resources are thoroughly assessed. Education about long COVID based on elements from a CBT framework [18] is provided. Tailored to the participant’s life, a personalized rehabilitation plan is created, consisting of home-based exercises to facilitate a combination of pacing and graded escalation of physical and cognitive activities. The home-based exercises have 3 focus areas: daily routine, physical activity, and cognitive training. The plan consists of weekly personal goals to guide the participant’s progress. Three video-follow-up consultations are given to support change and to encourage participants to adjust personal goals in step with recovery within the 3 focus areas. Supported by the physiotherapist, the personalized goal setting is then verbalized and adjusted during the follow-up consultations. In total, 4 consultations are given within 6‐8 weeks. Voluntarily, the participants can download the *My COVID Rehabilitation* mobile app as a supplement, where the goals can be noted and adjusted.

Textbox 1. **Overview of the content of the individual study intervention.**

- Part 1: Education about long COVID and coping strategies 
  - Session 1: Physical consultation (1.5 h) where symptoms, function in everyday life, sustaining factors and resources are mapped out. Provide education about long COVID based on elements of cognitive behavioral therapy. Information about treatment recommendations emphasizing adjustments of behavior. Tailored goal-setting work based on the 3 focus areas: daily routine (eg, sleep rhythm), physical activity (eg, housework or run), and cognitive exercises (reading comics).
  - *Home assignment:* To implement behavioral change according to participants’ personal goals, voluntarily noted in the *My COVID Rehabilitation* mobile app.
- Part 2: Behavioral change and home exercises
  - Session 2 and session 3: Video follow-up (60 min). Current status and review of home exercises after previous sessions. Tailored education about how adjustments in behavior may gain a positive health effect. Reflect on goal achievement and adjustments of goals.
  - *Home assignment*: Participants are encouraged to continue to work on their personal goals, voluntarily noted in the *My COVID Rehabilitation* mobile app.
- Part 3: Summary and further plan
  - Video follow-up (60 minutes). Current status and review of home exercises after previous session. Continued tailored education about how adjustments in behavior may gain a positive health effect. Reflect on goal achievement and adjustments of goals. Discussion about how to maintain and secure further progress.
  - *Home assignment:* Participants are encouraged to continue to work on their tailored personal goals, voluntarily noted in the *My COVID Rehabilitation* mobile app.

### Data Collection

Questionnaires will be sent to participants by email and captured by REDCap. The questionnaires are web-based and answered online. Reminders will be sent, if needed, by email once, SMS text messaging twice, and phone call once to improve adherence. If the participants have any questions regarding the questionnaires, they will be offered help by the research coordinator. The questionnaires will capture patient-reported outcome measurements at baseline and at 3, 6, and 12 months, including EQ-5D-5L index, EQ-5D-5L VAS, Chalder Fatigue Scale, number of symptoms, work participation, and feasibility of mobile app. At baseline, the patient also reports demographic data and relevant background information. Data is stored securely, and only the research team has access to the data. An overview of the measures and timepoints for assessments is presented in Table 1.

**Table 1.** Outcome measurements.

Type of outcome	Measurement	Timepoints
Health-related quality of life		
Primary outcome	EQ-5D-5L index [43]	6 mo
Secondary outcome (1)	EQ-5D-5L index [43]	0, 3, 12 mo
Secondary outcome (2)	EQ VAS^a^ [43]	0, 3, 6, 12 mo
Number of and grading of symptoms		
Secondary outcome	Self-completed questionnaire	0, 3, 6, 12 mo
Work participation		
Secondary outcome	Self-completed questionnaire	0, 3, 6, 12 mo
Fatigue		
Secondary outcome	Chalder Fatigue Scale (11 items) [44]	0, 3, 6, 12 mo
Utility and degree of mobile app usage		
Secondary outcome	Self-completed questionnaire	3, 6, 12 mo
Neuropsychological function		
Secondary outcome	Assessment from 4 cognitive domains: Episodic memory [45-47] Working memory: [48] Executive functions: [49,50] Processing speed [51]	0, 6 mo

a VAS: visual analog scale.

### Primary Outcome: HRQoL Measured With EQ-5D-5L

The primary outcome is HRQoL at the 6-month follow-up, assessed using the well-known EQ-5D-5L that gives a total index score [43,52]. EQ-5D-5L is a validated generic scale [52] and has also been applied and shown to be responsive to long COVID [37,53,54]. The 5 dimensions include mobility, self-care, usual activity, pain and discomfort, and anxiety and depression. Each dimension is assessed with 1 question, which has 5 levels of severity (“no,” “slight,” “moderate,” “severe,” and “extreme” problems). The Norwegian score has a range from −0.453 to 1, where 0 equals death and 1 is perfect health [55].

### Secondary Outcomes

#### Demographics

At baseline, demographic data are reported, including height, weight, time since infection, number of vaccines, medication, comorbidities, and highest level of education and occupational status.

#### Health-Related Quality of Life

HRQoL measured by the EQ-5D-5L index at 0, 3, and 12 months and the EQ visual analog scale (VAS) are part of the secondary outcomes. EQ-VAS instructs respondents to rate their overall health on a scale from 0 (the worst imaginable health) to 100 (the best imaginable health). This provides a global assessment of health status [56].

#### Fatigue

Fatigue is investigated using the Chalder Fatigue Scale as part of the questionnaire package. The fatigue scale is valid and reliable as a generic self-administered questionnaire for measuring the extent and severity of physical and mental fatigue. Each of the 11 items is answered on a 4-point scale ranging from asymptomatic to maximum symptomology [44], which are summed to a total score ranging from 0 to 33. The fatigue scale has previously been used in research on long COVID [57,58].

#### Symptoms

Symptom burden is explored through the presence of 13 common symptoms of long COVID (binary): fever, tingling, headache, dizziness, palpitations, nausea, cough, vomiting, diarrhea, altered taste and smell, sleep disturbances, chest pain, and muscle and joint pain. Participants also rate the severity of dyspnea and feeling of depression on a 4-point scale. The questions have previously been used in COVID research and have been shown to detect change [59].

#### Work Participation

Absence from work will be recorded as yes or no (binary) questions. In addition, the number of days of absence from work and the percentage of the absence are self-reported.

#### Mobile App

App use and perceived usefulness of the mobile app will be registered. Participants will be asked if they have used the mobile app. If yes, they will rate the frequency and usefulness on a 4-point scale.

#### Health Care Services

Participants will be asked to report contacts with the health care services and any use of medication.

### Neurocognitive Functioning

A comprehensive neuropsychological assessment will be conducted at baseline and repeated at 6 months to evaluate 4 key cognitive domains. The following measures from standardized tests will be used to measure aspects of the 4 selected cognitive domains.

1. Episodic memory: the average score from the total number of correct words recalled in the delayed recall trial of the Rey Auditory Verbal Learning Test [45,46] and the Rey Complex Figure Test [47].
2. Working memory: average score from the Letter-number-sequencing and Spatial Span subtests of the Wechsler Memory Scale-III [48].
3. Executive functions: average completion time for the trial 1-5 of the Trail Making Test and the Inhibition trial of the Color-Word Interference Test, both from the Delis-Kaplan Executive Function System [49,50].
4. Processing speed: average reaction time from the Conners Continuous Performance Test, 3rd edition [51].

Raw test scores are standardized using age-corrected published population norms. When available, published normative data from Scandinavian populations are prioritized. Scores will be reversed where necessary (reaction time) to ensure that higher scores consistently indicate better performance. Each assessment session will last 90 minutes, and the tests will be administered by a psychologist or trained health care personnel under supervision.

### mHealth Supplement: COVID Rehabilitation Mobile App

As part of this study, and at the request of our user representative, a mobile app was developed. The app has been named *My COVID Rehabilitation* (Norwegian: *Min COVID Rehabilitering*). Both groups will be invited to download the mobile app. The mobile app is a digital pamphlet with relevant information, including the possibility to make personal notes and goals based on activities from the 3 focus areas. Users will receive a daily reminder of their goals. After completing the activity, a simple digital reward is provided. The mobile app is available on the App Store and Google Play, and only the participant will have access to personal notes and goals in the mobile app on their phone. The mobile app has been developed in collaboration with user representatives, the multidisciplinary health care team at UNN, and system developers at Information and Communication Technology, Northern Norway Regional Health Authority. In the development process, feedback was given at several time points and adjustments made on both content, phrasing, and visual presentation.

### Explorative Outcomes

This trial is embedded in ordinary clinical care at UNN with the last author (M Wilhelmsen) as project leader. At the same time, the study is part of a national research collaboration called ReCover. Participants are therefore invited to participate in voluntary supplement investigations at baseline and at 6-month follow-up. Written informed consent, including the collection, storage, and use of participant data, is obtained at the first visit. Explorative investigations of immunological, inflammatory, and metabolic changes of markers in plasma/serum and whole blood RNA and EEG/MRI are done to explore changes in brain volume, density of white matter fiber tracts, and brain activity. The ReCover study will explore if immune profile and changes in the brain are modulated by the intervention. Details on these supplementary investigations will be provided in other articles from the ReCover research group.

### Statistical Analyses

#### Sample Size

Sample size justification was made in collaboration with a statistician. Due to the lack of studies with a similar design, we could not have completely precise estimates of the between-group difference in EQ-5D-5L index and standard deviation. We based our sample size estimation on a study by Espinoza-Bravo et al [37] that closely resembled ours. Espinoza-Bravo et al [37] compared 2 interventions consisting of home-based exercise with digital follow-ups in both groups. They found a mean difference between groups of (SD) 0.06 on EQ-5D-5L index and a standard deviation (SD) of 0.1 at the end of the intervention. There is no established minimal clinically significant difference (MCID) for the EQ-5D-5L index in long COVID [17]. However, for a respiratory condition (chronic obstructive pulmonary disease), the estimated MCID is 0.05 [60], and this has been used in previous research in long COVID [37]. In stroke rehabilitation, the MCID for the EQ-5D-5L index is found to be 0.10 [61]. In our study, we expect a higher mean difference (SD 0.07) in the EQ-5D-5L index between groups than in the study by Espinoza-Bravo et al [37] because we have a larger difference in treatment design. We also consider that the difference of 0.07 could be clinically significant and therefore based the sample size calculation on a mean difference of 0.07 and an SD of 0.1. We have a one-sided hypothesis where we assume that the intervention group will have a higher EQ-5D-5L index than the control group after 6 months. With a 15% (9/62) expected dropout, a power of 80%, and a *P* value of .05, the required sample size is estimated to be 31 participants in each group. The power estimation was run in SAS Enterprise Guide 8.3 using the POWER procedure. Other RCTs in populations with long COVID have demonstrated differences between groups with similar sample sizes [62,63].

#### Data Analyses

The analysis plan was made in collaboration with a statistician. The primary outcome, between-group difference in EQ-5D-5L index score at 6 months follow-up, will be analyzed by a statistician blinded to the intervention groups. Prior to statistical analysis, we will run an exploratory analysis of the outcome to check for data distribution and the presence of outliers, and to check for associations between the outcomes and covariates. To explore the effect of improved HRQoL, we will use linear models adjusted for baseline HRQoL, age of patients, and gender. The response variable in the model will be HRQoL at the 6-month follow-up. The linear model will be checked using common diagnostic tools (eg, distribution of residuals and influential observations). We will also run an exploratory analysis of the secondary outcomes. Next, because secondary outcomes will be measured repeatedly at 0, 3, 6, and 12 months, we will apply statistical models that are suitable for longitudinal data (eg, generalized estimating equation or mixed models class). Common model diagnostics will also be used for secondary outcomes.

Results for the primary outcome analysis were reported as linear regression coefficients with 95% confidence interval and *P* value. The standard cut-off of *P*<.05 will be used for statistical significance levels. We will evaluate if the difference is clinically and statistically significant. For secondary outcomes, we will report appropriate effect measures with 95% CIs.

For neurocognitive function, to facilitate comparison across cognitive domains, all scores will be converted to *z* scores (*z* = (x − μ)/σ). Each individual cognitive test score will be described by mean and SD, and the cutoff for cognitive impairment on any test or domain score will be set to 1 SD or less from the normative mean.

We will analyze significant between-group differences in cognitive domains at baseline to ensure comparability between the intervention and the control groups. We will also examine the mean changes in the 4 cognitive domains by calculating each participant’s average change in z-scores from T1 to T2 for each domain. These change scores are then compared between the intervention and control groups using significance testing and effect sizes [partial eta-squared (η²)]. Additionally, we will compute the Reliable Change Index (RCI) for each participant in both groups to categorize individual performance as stable, deteriorating, or improving based on clinically significant individual changes defined by the RCI. The RCI will be calculated using test-retest reliability estimates from published, normative samples [64].

### Ethical Considerations

The Regional Committee for Medical and Health Research Ethics in northern Norway (number 587293) approved this study based on protocol version 5, April 12, 2024.

The study has been registered at ClinicalTrials.gov under the identifier NCT06085911. This study will be conducted according to the ethical principles of the Declaration of Helsinki (2013) [65]. Written informed consent will be obtained from all included participants before baseline assessments. Consent and personal data are stored using a linkage key with deidentified data on a secure, closed research server. All personal data will be deidentified during analysis and dissemination of results. Participants do not receive any compensation for participating in the project, and they can unconditionally withdraw at any time.

## Results

The data collection (October 2023-December 2025) is conducted with grants from KlinBeForsk (grant 34476). Recruitment of participants and data collection started in November 2023. The data collection, including 12 months of follow-ups for all participants, is estimated to finish in November 2025. Recruitment of 62 participants was completed in October 2024. Results are expected to be published in a scientific journal in 2025 or 2026.

## Discussion

The present study investigates the effectiveness of tailored individual follow-ups versus a one-day group course in patients suffering from long COVID. Both groups are based on elements of CBT. Individual follow-ups with home-based exercises focusing on graded activity are expected to be more effective than a one-day group course. The main aim is to explore if the study intervention has better results of HRQoL at 6 months follow-up compared to a one-day group course. Other secondary outcomes will also be explored at different time points. Both intervention groups will be recommended to use the mobile app *My COVID Rehabilitation* as a supplement to the intervention for self-management of personal goals.

A strength of this study is that it is embedded in ordinary clinical care. This strengthens external validity. Further, this is a single-center study, and the lack of blinding for group allocation is considered a limitation. The inclusion age is set to 18‐65 years as one of the outcome measures in the study is return to work, which excludes older people and might therefore be a limitation. Long COVID is a complex condition, and therefore, both groups receive treatment combining several elements. The study will therefore not be able to identify which of the treatment elements were effective. Also, the study cannot conclude about the effectiveness of the mobile app as it is a voluntary element in both groups. The main outcome, EQ-5D-5L index, is recognized as a generic HRQoL measure and has proven to be responsive in long COVID populations [37,53]. Recent studies have questioned the instrument’s ability to capture fatigue as a particularly dominant symptom [66]. However, to compensate for this potential weakness, the Chalder Fatigue Scale, which is a more specific measure of subjective fatigue, is also included as an outcome measure.

Rehabilitation RCTs in patients with long COVID are limited and show a heterogeneity in both treatment content, treatment approach, and outcome measures, making it difficult to conclude the effectiveness [3,15,17]. Common to many interventions is that they provide clinical follow-ups over time, often 6 to 12 weeks [3,15,22,35]. It is still unclear how treatment content should be combined and which modality is most effective. We argue that long COVID is a complex condition and thereby may demand treatment with more than 1 focus area and individual follow-ups to reduce the impact of long COVID. This study will provide knowledge about rehabilitation interventions in long COVID, their effectiveness, and whether individual follow-ups are better than a 1-day group course. This study has potentially important implications for patients with long COVID, health services, and society. Individual follow-ups are expected to lead to increased adherence in terms of the individual rehabilitation plan within the 3 focus areas and thereby achieving better results in terms of less symptom burden, increased function, work participation, and lower costs for society. Additionally, the study will generate important knowledge about neuropsychological function and digital self-management tools in long COVID rehabilitation.

## Supplementary material

10.2196/74113 Multimedia Appendix 1 Consent form.

10.2196/74113 Checklist 1 SPIRIT checklist.

## Abbreviations

CBT: cognitive behavioral therapy
CFS: chronic fatigue syndrome
HRQoL: health-related quality of life
ME: myalgic encephalomyelitis
RCT: randomized controlled trial
REDCap: Research Electronic Data Capture
SPIRIT: Standard Protocol Items: Recommendations for Interventional Trials
UNN: University Hospital of North Norway
WHO: World Health Organization
