# Supplementary material for: Tailored Individual Follow-Ups Versus a One-Day Group Course in Patients With Long COVID (Post– COVID-19 Condition): Protocol for a Randomized Controlled Trial
Source: JMIR Res Protoc. 2026 Jan 5;15:e74113. doi: 10.2196/74113 (PMC12768394; doi:10.2196/74113)
Supplement: Multimedia Appendix 1 [file resprot-v15-e74113-s001.pdf]

Request for participation in a research project on long-term symptoms after Covid-19

Version 1.4, 2023

What are you being asked to consent to?

You are being asked to participate in a research project that aims to study how different rehabilitation approaches affect long-term symptoms after Covid-19. We will explore the impact on symptoms, quality of life, work, and neuropsychological functioning.

The study is part of a national research collaboration. The purpose of the study is to determine which rehabilitation method is most effective.

We are asking you because you have been referred to the University Hospital of North Norway (UNN) due to long-term effects after testing positive for Covid-19 and are experiencing symptoms we wish to investigate further.

The University Hospital of North Norway is responsible for the study. Our partners are St. Olav's Hospital, Haukeland University Hospital, and Oslo University Hospital.

What does the project involve?

Based on random selection (randomization), half of the participants will receive a one-day course on how to manage long-term effects after Covid-19. The other half will receive individual follow-up with a therapist at the Rehabilitation Department at UNN in Tromsø, a total of four consultations. Each consultation lasts between 60 to 90 minutes. Both groups will receive digital tools with useful information/resources for coping.

You will be asked to complete a digital questionnaire on the first day of treatment, and again after 3, 6, and 12 months. The initial questionnaire takes about 30 minutes to complete, while the follow-ups take about 10 minutes each.

In the project, we will also collect and register information about you. We will record medical information relevant to long-term effects of Covid-19. This information will come from you or your medical records, including vaccination history, medications, height, weight, other illnesses, X-rays, treatments, and lab results.

A neuropsychological assessment will also be conducted at the start and after 6 months. This will include tests of your memory, attention, and ability to shift focus. The tests will be performed at UiT – The Arctic University of Norway, during the same visit as your appointment at UNN. The neuropsychological testing takes 1 to 2 hours.

Possible benefits and drawbacks

We do not yet know what the best rehabilitation approach is for long-term effects after Covid-19. Therefore, there is no specific disadvantage associated with being assigned to either group. If you choose not to participate, you may still attend the one-day course if you wish.

By participating, you will contribute to important research on how different types of rehabilitation can impact prognosis.

If you choose to participate, you will spend some time completing simple questionnaires and undergoing neuropsychological testing.

Voluntary participation and withdrawal of consent

Participation in the project is voluntary. If you wish to participate, sign the consent form on the last page. You may withdraw your consent at any time without giving a reason. This will not affect your further treatment.

If you withdraw from the project, you may request the deletion of collected samples and data, unless the data has already been included in analyses or used in scientific publications.

Contact information:

If you later wish to withdraw, experience discomfort with the study, or have questions about the project, please contact:

- Research coordinator Marte Wilson
  Phone: 77669642
  Email: marte.wilson@unn.no
- Principal investigator Maja Wilhelmsen
  Phone: 77627834 / 99001559
  Email: maja.wilhelmsen@unn.no

Questions regarding privacy can be directed to: personvernombudet@unn.no

What happens with your information?

The information recorded about you will only be used as described in the project’s purpose. You have the right to access the data registered about you, and the right to correct any errors in the recorded data.

All data will be stored without your name, personal ID number, or other directly identifying information. A code will link you to your data via a name list. This means the data is de-identified. The list linking your name to the code will be stored on a research server at the University Hospital of North Norway and only accessible to study staff.

We may collect additional information such as medication use, sick leave, vaccination status, and time of positive Covid test from your medical record or public registries like the vaccination register, prescription register, and sick leave register.

Publishing the results is a necessary part of the research process and will be done using aggregated data, making it extremely unlikely that you will be identifiable.

Any expansion in data use can only occur with approval from the Regional Committee for Medical and Health Research Ethics (REK). As part of the study, your data may be shared with other research projects on long-term effects of Covid-19 in Norway, the EU, or the UK. UNN will ensure that data transfers comply with Norwegian law and data protection regulations (GDPR). The code linking you to personally identifiable information will not be shared.

Insurance

All participants in the study are covered under the Norwegian Patient Injury Act (LOV-2001-06-15-53).

Follow-up study

If we plan a follow-up project, we may contact you again.

Funding

The study is funded by a Clinical Treatment Research Program (KlinBeForsk), which is financed by the Ministry of Health and Care Services and the regional health authorities, as well as funds from Helse Nord.

Travel to and from Tromsø will be reimbursed through the Norwegian patient travel system. No other compensation is provided to participants.

Approval

The project is approved by the Regional Committee for Medical and Health Research Ethics (REK), reference number: **REK 587293**.

The processing of your personal data has a legal basis in article 6 no. 1 letter e and article 9 no.2 letter j of the EU General Data Protection Regulation (GDPR), and Sections 8 and 9 of the Personal Data Act – for research purposes in the public interest.

Your consent is obtained in accordance with the Health Research Act, to ensure transparency, participation, and predictability in the processing of your data.

You have the right to file a complaint about how your data is handled with the **Norwegian Data Protection Authority (Datatilsynet).**

Consent Declaration

I consent to participate in the study and to the use of my personal data as described.

By signing below, I confirm that I have received information about the study, that any questions I had have been answered, and that I have received a copy of the information sheet.

Place and date

Participant's signature

Participant's name in block letters

Place and date

Study staff signature

Study staff name in block letters
